# Adherence to Oseltamivir Guidelines during Influenza Pandemic, the Netherlands

**DOI:** 10.3201/eid1803.111351

**Published:** 2012-03

**Authors:** Esther H. Fietjé, Daphne Philbert, Erica C.G. van Geffen, Nina A. Winters, Marcel L. Bouvy

**Affiliations:** Author affiliation: Utrecht University, Utrecht, the Netherlands

**Keywords:** the Netherlands, viruses, oseltamivir, pandemic influenza, viruses, antiviral agents, guideline adherence, medication adherence, influenza A virus

**To the Editor:** In the Netherlands, the outbreak of pandemic influenza A (H1N1) 2009 led to a 100-fold increase from 2008 in prescriptions for the antiviral neuraminidase inhibitor oseltamivir (*1*). The guidelines for prescribing oseltamivir during the 2009 pandemic were adapted throughout the year. After August 7, prescribers were advised to restrict prescriptions to patients with influenza symptoms plus 1 additional risk factor (*2*) (Table).

**Table:** Reported risk factors of patients with and without influenza symptoms who were dispensed a prescription for oseltamivir, the Netherlands, 2009–10

Risk factor	No. (%) patients*		
	Total, n = 300	Influenza symptoms, n = 223	No influenza symptoms, n = 77
Chronic condition	211 (70.3)	154 (69.1)	57 (74.0)
Chronic respiratory disease	127 (42.3)	98 (43.9)	29 (37.7)
Lower immune resistance caused by illness or medical treatment	76 (25.3)	50 (22.4)	26 (33.8)
Cardiovascular disease	52 (17.3)	34 (15.2)	18 (23.4)
Diabetes	44 (14.7)	28 (12.6)	16 (20.8)
Renal disease	10 (3.3)	5 (2.2)	5 (6.5)
Other	125 (41.7)	86 (38.6)	39 (50.6)
Age >60 y	66 (22.0)	35 (15.7)	31 (40.3)
Age <2 y	36 (12.0)	35 (15.7)	1 (1.3)
Regular patient contact by health care worker	22 (7.3)	15 (6.7)	7 (9.1)
Pregnancy	5 (1.7)	2 (0.9)	3 (3.9)
No. risk factors			
0	44 (14.7)	34 (15.2)	10 (13.0)
1	137 (45.7)	111 (49.8)	26 (33.8)
2	72 (24.0)	53 (23.8)	19 (24.7)
>3	47 (15.7)	25 (11.2)	22 (28.6)

*Percentages may total >100% because of rounding.

Community pharmacists dispensed oseltamivir as a 5-day course of sachets produced exclusively for the Dutch government program and documented all prescriptions. Our objective was to assess whether oseltamivir dispensed through community pharmacies was prescribed according to the national guideline for the pandemic virus and to investigate how patients used oseltamivir. The Institutional Review Board of the Division of Pharmacoepidemiology and Clinical Pharmacology of Utrecht University approved the study.

Pharmacists in 19 pharmacies belonging to the Utrecht Pharmacy Practice Network for Education and Research (UPPER) selected all patients who had filled a prescription for oseltamivir during May 1, 2009–February 8, 2010. These patients were contacted by phone and, after giving consent, completed a structured questionnaire. The questionnaire contained questions about potential risk factors, the reason for receiving the oseltamivir prescription (influenza symptoms or other reasons), and whether the oseltamivir course was started and completed.

Of the 630 patients eligible for contact, 361 (57.3%) completed the questionnaire. To assess whether the current guidelines were adhered to, because of the changes in policy throughout the year, we analyzed only the 300 respondents who had filled the oseltamivir prescription at the height of the pandemic, i.e., after August 7, 2009.

A total of 156 (52.0%) participants were female patients; most participants were 18–59 years of age. Of the 212 patients >18 years of age, education level was available for 195; of these, 55 (28.2%) had a low education level, 94 (48.2%) a middle education level, and 46 (23.6%) a high education level.

Of the 300 respondents, 111 (37.0%) received a prescription while they did not meet guideline criteria (Table). They had risk factors but did not experience influenza symptoms (67 [22.3%] of all respondents); had influenza symptoms but not risk factors (34 [11.3%]); or had neither influenza symptoms nor any risk factors (10 [3.3%]).

Compared with respondents who had a low education level, respondents >18 years of age who had a middle or high education level were 2× more likely to receive an oseltamivir prescription that was not in accordance with guideline criteria (odds ratio 2.20; 95% CI 1.12–4.32). Sex and age were not associated with the likelihood of receiving off-guideline oseltamivir.

Of the 189 respondents who received oseltamivir in accordance with guideline criteria, 184 (97.4%) started treatment and 167 (90.8%) completed the oseltamivir course. Of the 111 respondents who received a prescription for oseltamivir that was not in accordance with guideline criteria, 62 (55.9%) started treatment, and 56 (90.3%) completed the course.

We showed that during the pandemic the guideline criteria were not met by nearly one third of patients who received an oseltamivir prescription. Patients with a higher education level more often received a prescription, suggesting that they are more informed or empowered than patients with a lower education level to request a prescription. Another explanation for the inadequate adherence to guideline criteria is that prescribers themselves were not immediately aware of the current criteria, possibly because of changes throughout the year.

In addition, in nearly half of instances in which guideline criteria were not met but in which oseltamivir was prescribed, the patients did not start the oseltamivir course. These prescriptions could have been used for stockpiling, which also occurred during the influenza A (H5N1) outbreak in 2005 (*3*). In the Netherlands, stockpiling did not lead to drug shortages, but in countries where oseltamivir is not reimbursed by the government, stockpiling might lead to problems with availability for patients truly in need of antiviral therapy but without the necessary means to acquire it.

The limited effect of oseltamivir on reducing disease duration, usually only shortening the duration by 1 day in healthy persons (*4*), the possibility of serious side effects (*5*), the possibility of the virus developing resistance to neuraminidase inhibitors (*6**,**7*), and the cost to health care of unnecessary prescriptions are reasons to strive for better adherence to prescribing guidelines. Prescribers need to be properly informed about current guidelines to reduce overprescribing caused by lack of knowledge. Furthermore, improving communication between prescribers and patients might help relieve patients’ concerns and increase awareness about the limited benefits of oseltamivir treatment in healthy persons.
